# Comment on Ombashi, van der Goes, Versnel, Khonsari, van der Molen: guidance to develop a multidisciplinary, international, pediatric registry: a systematic review, Orphanet Journal of Rare diseases, 2023

**DOI:** 10.1186/s13023-024-03123-y

**Published:** 2024-04-17

**Authors:** Kristina Klintö, Magnus Becker

**Affiliations:** 1 Department of Clinical Sciences in Lund, Lund University, Lund, Sweden; 2 Department of Otorhinolaryngology, Skåne University Hospital, Malmö, Sweden; 3 Department of Clinical Sciences in Malmö, Lund University, Malmö, Sweden; 4 Department of Plastic and Reconstructive Surgery, Skåne University Hospital, Malmö, Sweden

**Keywords:** Registry, Cleft lip and palate, Surgery, Orthodontics, Speech

## Abstract

Recently, Ombashi et al. published a systematic review aiming to identify the pitfalls in the development and implementation as well as factors influencing long-term success of a multidisciplinary, international registry for cleft care on a global scale. The purpose of this letter to the editor is to highlight that the review failed to include the Swedish quality registry for patients born with cleft lip and palate, which fulfils the inclusion criteria. The Swedish cleft lip and palate registry is multidisciplinary, has a high coverage and reporting degree, and most outcome measures have been checked for reliability and validity. It is regularly used for open comparisons between treatment centers. Several research studies have been published based on the Swedish cleft lip and palate registry, and more are ongoing. The information we provide about the Swedish cleft lip and palate registry complements and expands the information of the results reported by Ombashi et al. in their research.

## Main text

Recently, Ombashi et al. [1] published a systematic review to provide a scientific basis for the conceptualization of a European wide registry for cleft lip and palate (CLP) care, by studying previous registry initiatives. The aim was to identify the pitfalls in the development and implementation as well as factors influencing long-term success of a multidisciplinary, international registry for cleft care on a global scale. The authors stated that “Studies were included if their primary or secondary aim was to describe the design and/or the methods to develop or maintain a multidisciplinary registry that involves pediatric patients or are specifically designed for pediatric patients”. Regional, national, and international registries were included.

Although we acknowledge the authors’ efforts, we write this letter to highlight that the review failed to include the Swedish quality registry for patients born with CLP, which fulfils the inclusion criteria [2]. The Swedish CLP Registry is a multidisciplinary registry, and data on children in Sweden with CLP, born from 2009 onwards, are included. At the first visit baseline data, such as cleft type (ICD-10 diagnosis), heredity, birth weight and additional deformities and/or syndromes, as well as pre-surgical treatment, are recorded. Data on surgical treatment are recorded continuously, and treatment outcome regarding dentofacial development and speech are recorded at follow-ups at 5, 10, 16 and 19 years of age. In addition, data on dentofacial development are recorded 1 year after orthognathic surgery, and data on babbling and speech at 18 months of age. All Swedish CLP centers are connected to the registry. The Swedish CLP Registry has a high coverage degree (above 90%), a high reporting degree for most variables, and most outcome measures have been checked for reliability and validity [2–5]. It is possible to make open comparisons between the Swedish CLP centers based on the Swedish CLP Registry, and these are published annually in the CLP Registry’s reports and online [6]. Several research studies have been published based on the Swedish CLP Registry [7–9], and more are ongoing. The information we provide about the Swedish cleft lip and palate registry complements and expands the information of the results reported by Ombashi et al. in their research.

## Data Availability

Not applicable.

## Abbreviations

CLP: Cleft lip and palate
